# The significance of arginase-1 expression in the diagnosis of liver cancer: A protocol for a systematic review

**DOI:** 10.1097/MD.0000000000019159

**Published:** 2020-02-28

**Authors:** Xuejiao Wang, Yifei Xu, Ruochong Wang, Ning Dai, Wei Zhang, Feng Li

**Affiliations:** Beijing University of Chinese Medicine Beijing, China.

**Keywords:** a systematic review, ARG-1, diagnosis, hepatocellular carcinoma, liver cancer, meta-analysis, protocol

## Abstract

**Background:**

Hepatocellular carcinoma (HCC) is the most common primary liver cancer. Pathologic distinction between HCC and intrahepatic cholangiocarcinoma (ICC) and metastatic adenocarcinoma can be challenging and sometimes requires immunohistochemical panels. Recently, arginase-1 (ARG-1) has been introduced for differentiation of these tumors.

**Methods:**

We will search Cochrane Library, PubMed, Embase, China National Knowledge Infrastructure through August 1, 2019, comprehensive collection studies about the diagnostic value of ARG-1 for HCC. Two reviewers will screen literature according to the inclusion and exclusion criteria, extract data, and assess the quality of included studies. Review Manager 5.3 and STATA 15.0 will be used to conduct the meta-analysis.

**Results:**

The review will provide a high-quality synthesis of current evidence of the diagnostic value of liver cancer. The results will be published in a peer-reviewed journal.

**Conclusion:**

We hope that the results of this study will provide significant evidence to assess the value of ARG-1 in differential diagnosis of HCC, ICC, and metastatic carcinoma of liver.

## 1 Introduction

Primary hepatocellular carcinoma (HCC) is a malignant tumor occurring in hepatocytes or intrahepatic bile duct cells, called HCC and intrahepatic cholangiocarcinoma (ICC), respectively.^[1]^ The most common type of primary liver cancer globally is HCC, followed by ICC.^[2]^ Liver cancer is the sixth most common incident cancer worldwide and the fourth most common cause of cancer death worldwide.^[3]^ The disease occurs frequently in underdeveloped countries such as Asia and Africa, also in China.^[1]^ As a country characterized by a high incidence of liver disease, it puts forward urgent requirements for screening, diagnosis, treatment and other prevention on us Chinese.

About 80% of HCCs occurs in the background of cirrhosis, while the remaining HCCs occurs in noncirrhotic livers.^[4]^ These patients are routinely screened by imaging at 6-month intervals for the detection of small (1–2 cm) lesions, in hopes of detecting cancer at an early and potentially curative stage.^[5]^ HCC and ICC tend to have malignant imaging features, however, the features are not sufficiently specific to permit noninvasive diagnosis.^[6,7]^ Biopsy of such lesions for definitive histologic diagnosis is frequently performed. Although in some cases the evaluation of liver lesions may be accomplished by routine hematoxylin-eosin (H&E) staining, it is also common to use ancillary tests to arrive at a definitive diagnosis. Koehne et al^[8]^ believes it is necessary to use at least a minimal panel of immunostains in all but the most classic cases in which it may be reasonable to rely strictly on H&E Morphology.

As the high sensibility and specificity, arginase-1 (Arg-1) seems the best choice of marker for liver tissue with sensitivities ranging from 83% to 100%. However, there are studies expressing different result. The sensibility of Arg-1 differ as the histological differentiation. Some studies suggest that, arg-1 is sensitive especially in high differentiation,^[9–20]^ while 1 newly study find a subset of well-differentiated HCCs are Arg-1 negative.^[21]^

Globally, HCC is the dominant histologic type of liver cancer in most countries accounting for approximately 80% of total cases. ICC is the second most common histologic type, accounting for approximately 15% of total cases.^[22]^ Though, the 2 have roughly the same clinical manifestations, ICC is different from HCC in etiology, pathogenesis, and treatment. A differential diagnosis between ICCs and HCCs is often challenging, particularly in cases of poorly differentiated carcinoma.

There is still such a fact, as we all know, in noncirrhotic patients, metastatic tumors to liver are more common. However, there still 7%-54% of HCC arising in noncirrhotic patients. This situation put another challenge on distinguish HCC from metastatic carcinoma.^[23]^ Yan et al^[20]^ found the specificity of arg-1 is 100% when examining only tumors that were morphologically similar to HCC. This exciting result can be doubtful for Chandan et al^[24]^ observed Arg-1 in 8 adenocarcinoma with hepatoid features and found 5 of them are Arg-1 positive. In the study of Radwan^[16]^ and Fujiwara,^[11]^ Arg-1 is positive in cases of pancreatic adenocarcinoma. What's more Bita^[12]^ demonstrated 1 gastric adenocarcinoma present intensity and diffuse straining for Agr-1. This is confusing indeed.

With all these situation in mind, there is renewed interest to explore the value of Arg-1 in differential diagnosis of HCC, ICC, and metastatic carcinoma of liver.

## 2 Outcomes

The primary outcome measures will be sensitivity, specificity, negative predictive value, positive predictive value, area under the curve. Additional outcome(s) have not been planned.

## 3 Materials

### 3.1 Standards

The protocol of the meta-analysis will be developed according to the Preferred Reporting Items for Systematic Reviews and Meta-Analysis for diagnostic test accuracy study and preferred reporting items for systematic reviews and meta-analyses protocols (PRISMA-P) guidelines. PRISMA-P is supplied in PRISMA checklist.

### 3.2 Ethical issues

Ethical approval is not required because this is a literature-based study based on previously aggregate data and will be no direct contact with individual patients.

### 3.3 Registration

Our meta-analysis protocol has been registered in the PROSPERO network with registration number: CRD42019147680.

But, we plan to make a little change in the review questions to which delete the contrast between HCC and combined hepatocellular-cholangiocarcinoma.

### 3.4 Data sources and search strategy

A comprehensive literature search will be conducted in Cochrane Library, PubMed, Embase, China National Knowledge Infrastructure regardless of language from their inception to August 1, 2019 using the following MeSH words: (Hepatocellular Carcinoma OR HCC OR Hepatoma OR Liver cancer OR hepatocarcinoma) AND (arginase I OR arg-I OR arginase 1 OR arg-1). Only electronic searches for studies will be performed. Furthermore, the reference lists of the identified articles obtained from the original search will be manually searched to identify additional relevant studies. The example search strategy in Table 1 (appendixed below References) will be used in PubMed. This search strategy will be modified and adjusted to the specific requirements of other databases.

**Table 1:**
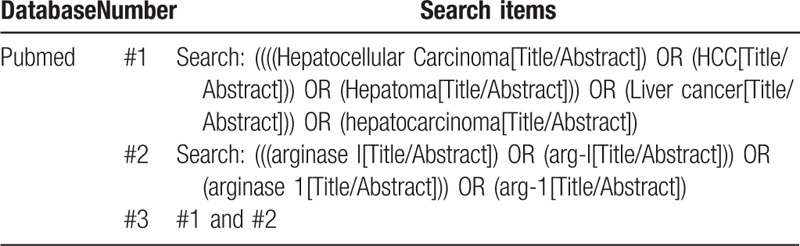
Preliminary search strategy for PubMed.

### 3.5 Types of studies

We will include both comparative studies (comparison of 2 or more index tests against the reference standard), and non-comparative studies (single index test against the reference standard). We will include diagnostic studies with prospective or retrospective case control, cohort, and cross-sectional studies.

### 3.6 Types of patients

Patients (aged 18 years or over) with HCC which has been confirmed on histology without the limitation of gender or race.

### 3.7 Types of interventions

This meta-analysis is not on interventional studies. The subjects are the hepatocarcinoma patients who had Arg-1 antibody been used for immunohistochemistry. The control group could be patients who have confirmed the presence of ICC, and metastatic carcinoma to liver.

### 3.8 Data collection and analysis

#### 3.8.1 Selection of studies

Two reviewers will search and screen independently. Before selection of studies, all reviewers must get trained to understand the purpose and process of the review. The results will be exported to the Endnote X8 and duplicate studies will be removed by it. Initially, we will screen and evaluation the titles and abstracts of studies, and select those likely to be of relevance to our systematic review.

**Figure 1:**
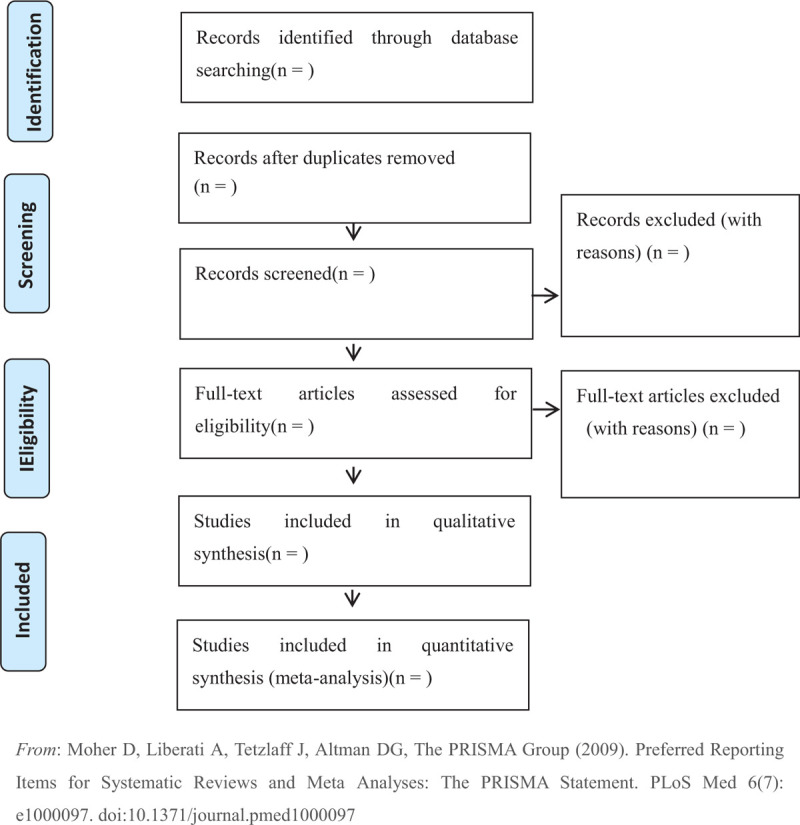
Flow chart of studies selection process.

In the second stage of selection, full texts will be examined if necessary. Any disagreements should be resolved through discussion to get a consensus and judged by an arbiter (Feng, Li). The study selection procedure is shown in the flowchart, see in Figure 1 (appendixed below References).

#### 3.8.2 Data collection and management

Two investigators will independently extract all data, and any discrepancies will be resolved by discussion. The following data from each eligible study will then be extracted: first author, year of publication, country of publication, study design, characteristics of enrolled populations (numbers of HCC cases and controls, baseline diagnosis, mean age, gender, if available), reference standard, biopsy method, indicators of diagnostic value (specificity, sensitivity, area under the curve, *P* value).

When the performance characteristics were not reported in detail, the information will be extracted from relevant graphs and tables. Or we will contact the corresponding author for more information as possible.

#### 3.8.3 Assessment of risk of bias in included studies

The quality of the included studies will be independently assessed using the quality assessment of diagnostic accuracy studies 2 (QUADAS-2)^[25]^ by 2 researchers. Any discrepancies arising will be resolved through discussion and consensus.

#### 3.8.4 Dealing with missing data

If the required data are not clear or not reported in clinical papers, we will contact with the original author of the studies for complete information via e-mail. If not successful, we will analyze available data to perform the outcome; Meanwhile, we will also assess the potential impact of missing data on the conclusion in the discussion.

#### 3.8.5 Assessment of publication bias

We would not evaluate publication bias because these methods are not appropriate for studies of diagnostic accuracy.

#### 3.8.6 Data synthesis

All analyses will be conducted with the appropriate software (for example Review Manager 5.3, STATA 15.0 as needed). We will base summary measures on the characteristics of the summary receiver operating characteristic curve (SROC), their predictive value as well as their likelihood ratio. For the SROC we will plot from each one of the included studies the sensitivity as a function of the false positivity (1-specificity). However, if we observe variation in the threshold used by the included studies to define a test positive, the summary sensitivity and specificity point of the test for a common threshold or several thresholds, referred as the average operating point, will be determined.

The general approach will be that suggested in Chapter 10 of the Cochrane Handbook for Systematic Reviews of Diagnostic Test Accuracy.

For all included studies, we will enter the data in the two-by-two tables into Review Manager 5 software (Review Manager 5), which will allow the sensitivities, specificities and their 95% confidence intervals to be presented in forest plots and receiver operating characteristic space. These presentations will be used to explore the included study results, focusing on the test threshold in common use in clinical practice. The interaction between test accuracy results, study characteristics, and methodological study quality will also be considered.

We will interpret the results and prepare a summary of results table using Chapter 11 of the Cochrane Handbook for Systematic Reviews of Diagnostic Test Accuracy as guidance.

When *I*^*2*^ < 50%, a fixed-effects model will be used to calculate the risk radio and mean difference. When *I*^*2*^ ≥ 50%, we will use a random-effects model to synthesize the data. If apparent clinical heterogeneity is demonstrated, the reviewers can carry out the subgroup or sensitivity analysis to explore heterogeneity source including clinical and methodology cause. On the contrary, we only perform descriptive analysis if meta-analysis is not applicable.

#### 3.8.7 Subgroup analysis

If appropriate, subgroup analyses will be conducted concerning different regions, biopsy methods, and the differentiation of liver cancer.

#### 3.8.8 Sensitivity analysis

To ensure the robustness of evidence, we will perform sensitivity analysis to assess the impact of studies with high risk of bias according to QUADAS 2. We will compare the results to decide whether studies with lower quality should be excluded on the basis of sample size, strength of evidence and influence on pooled effect size.

#### 3.8.9 Ethics and dissemination

Ethical approval is not necessary because data used in our study are not linked to individual patient data. Also, the findings will be disseminated through a peer-review publication.

## 4 Discussion

Diagnosis marks the beginning of any successful therapy. HCC, ICC, and metastatic adenocarcinoma to the liver are different in etiology, pathogenesis, and treatment, even in the syndrome differentiation in traditional Chinese medicine. Results from this systematic review will inform clinical practice and research on ARG-1 and liver cancer. To the best of our knowledge, this is the first systematic review that will examine the expression of ARG-1in the diagnosis of liver cancer. Gaps in the literature will be identified to provide suggestion for future

However, this review still has some limitations. Due to searches barriers, only electronic searches will be applied. Besides, different countries and the subjectivity of immunohistochemistry may run risk of heterogeneity.

## Author contributions

**Conceptualization:** Xuejiao Wang.

**Methodology:** Ning Dai.

**Project administration:** Ruochong Wang, Wei Zhang.

**Writing – original draft:** Xuejiao Wang.

**Writing – review & and editing:** Yifei Xu, Ruochong Wang.
